# Life Cycle Assessment of Microalgae-Based Products for Carbon Dioxide Utilization in Thailand: Biofertilizer, Fish Feed, and Biodiesel

**DOI:** 10.12688/f1000research.159019.1

**Published:** 2024-12-09

**Authors:** Adeel Rafiq, Cameron Morris, Abigail Schudel, Shabbir Gheewala

**Affiliations:** 1 The Joint Graduate School of Energy and Environment, King Mongkut's University of Technology Thonburi, Bangkok, 10140, Thailand; 2 Center of Excellence on Energy Technology and Environment (CEE), Science, Research and Innovation, Ministry of Higher Education, Bangkok, 10400, Thailand; 3 Institute for the Environment, , Chapel Hill, NC, USA, University of North Carolina at Chapel Hill, Chapel Hill, USA; 4 University of North Carolina, Department of Environmental Sciences and Engineering, Gillings School of Global Public Health, NC, USA

**Keywords:** carbon capture, carbon utilization, photobioreactor, Chlorella vulgaris, aquafeed, life cycle assessment, microalgae

## Abstract

**Background:**

Microalgae-based products offer a sustainable solution for food, fuel, and agricultural inputs, presenting environmental benefits and economic opportunities. A comprehensive assessment is needed to understand their potential in supporting sustainability goals, considering the complex interplay between production methods, energy sources, and environmental impacts.

**Methods:**

This study evaluated the environmental impacts of three microalgae-derived products – biodiesel, fish feed, and biofertilizer – through a comprehensive life cycle assessment. Nine scenarios were explored comparing three electricity profiles (current Thai mix, 50% renewable/50% current mix hybrid, 100% renewable) across the three products. The assessment evaluated environmental impacts and potential economic benefits of transitioning to these microalgae-based alternatives.

**Results and discussion:**

All products demonstrated potential for significant environmental benefits under increased renewable energy scenarios. Fish feed consistently exhibited the lowest environmental impacts across all categories examined, showing substantial improvements with increased renewable energy use. With an annual demand of 0.4 million tonnes, fish feed could generate USD 560 million in revenue and reduce CO _2_ emissions by 1.1 million tonnes. Fulfilling the projected biodiesel demand of 4,015 million liters per year through microalgae production could yield approximately USD 3.5 billion in revenue and reduce CO _2_ emissions by 30 million tonnes compared to conventional fossil-based diesel. Additionally, algal biofertilizer production could meet a 5 million tonnes annual demand, offering USD 2 billion in revenue while reducing CO _2_ emissions by 6 million tonnes yearly. Collectively, these products could offset 37 million tonnes of CO _2_, representing about 14% of Thailand’s total CO _2_ emissions, contributing significantly to the country’s Nationally Determined Contribution (NDC) target of 20-30% greenhouse gas emissions reduction.

**Conclusion:**

Transitioning to microalgae-based products could transform the aquaculture, energy, and agricultural sectors, potentially supporting the national climate change mitigation goals, if implemented.

## 1. Introduction

Microalgae has emerged as a promising candidate for carbon utilization amid growing concerns about climate change and the need to reduce carbon dioxide (CO _2_) emissions ( Rosental et al., 2020). Rising levels of greenhouse gases, especially CO _2_, have posed major challenges to global sustainability and climate stability in recent decades, largely due to continued reliance on fossil fuels for energy and industry. According to emissions trends, CO _2_ and other greenhouse gases will result in exceeding the 1.5 °C warming goal set by the Paris Agreement ( Adun et al., 2024). As a result, carbon dioxide removal will be necessary to meet global and national climate goals. Carbon capture technology has been developed to abate carbon emissions, however the path to utilization still remains unclear. Microalgae may offer a sustainable approach to help address this issue ( Shuba and Kifle, 2018; Sun et al., 2018).

Additionally, microalgae products could present a solution for the growing global population’s increasing demand for food, energy, and agricultural products ( Braun and Colla, 2023). Microalgae has fast growth rates, high photosynthetic efficiency, and can sequester CO _2_ much more quickly than traditional crops ( Pignolet et al., 2013; Yahya et al., 2020). Their high surface area-to-volume ratio also facilitates efficient gas exchange with the surrounding environment, enhancing CO _2_ uptake ( Bhola et al., 2014). Furthermore, microalgae thrive in CO _2_-rich conditions, making it well-suited to capture emissions directly from industrial sources like power plants and cement factories ( Yahya et al., 2020). To harness these advantageous properties of microalgae for effective carbon utilization and product development, different cultivation methods have been developed and refined over the years. Microalgae cultivation methods primarily include open raceway ponds and closed photobioreactors. Open raceway ponds are shallow, circulating channels that offer low capital and operational costs but are susceptible to contamination and weather fluctuations ( Jorquera et al., 2010; Richardson et al., 2012; Skjånes et al., 2016). Closed photobioreactors, such as tubular, flat panel, or column systems, provide better control over growth conditions, higher productivity, and reduced contamination risk, albeit at higher costs ( Chisti, 2007; Jorquera et al., 2010; Richardson et al., 2012).

Recent years have witnessed a surge of interest in products derived from microalgae, leading to numerous life cycle assessment (LCA) studies that evaluate their environmental sustainability and impacts. A number of studies have examined the environmental profile of algae-derived biofuels. Clarens et al. (2011) found that algae systems produce significantly more vehicle kilometers traveled per hectare than terrestrial crops, but their environmental performance per kilometer is mixed. Their study emphasized the importance of nutrient procurement in algae cultivation and noted that using recycled CO _2_ from flue gas significantly improved the energy return on investment compared to virgin commercial CO _2_ ( Clarens et al., 2011). However, they noted that flue gas capture for algae cultivation is not yet viable at industrial scale. Fenton et al. (2014) conducted an LCA assessing algal biofuels’ potential to replace petroleum fuels in Bangkok, Thailand. The study found that algal biofuels could replace less than 1% of Bangkok’s petroleum fuel use due to limitations in nutrient availability from municipal wastewater. Additionally, algal biofuels had higher environmental impacts than conventional fuels across all categories assessed ( Fenton et al., 2014).

Similarly, the LCA study by Bradley et al. (2023) revealed that microalgae-derived biodiesel, when accounting for infrastructure, currently has higher greenhouse gas emissions than petroleum diesel. To match the environmental performance of petroleum diesel, significant improvements in microalgae productivity are necessary. The study underscored the importance of optimizing energy sources and feedstocks, such as yeast, to enhance the sustainability of microalgae-based biofuels ( Bradley et al., 2023). Schade and Meier (2020) conducted an LCA to evaluate the environmental impacts of microalgae cultivation for food. They found that microalgae cultivation in a cold-weather climate can be sustainable, and a longer cultivation season is preferable even with reduced productivity. Similarly, Smetana et al. (2017) investigated microalgae as a potential solution to protein shortages in Europe. Their LCA revealed that current microalgae cultivation methods have a higher environmental impact than traditional protein sources. However, they identified promising alternatives, such as using heterotrophic cultivation and food waste as feedstock, which could significantly reduce the environmental impact and make microalgae a sustainable protein source ( Smetana et al., 2017). High moisture extruded products made from heterotrophically cultivated *Chlorella vulgaris* showed lower environmental impacts than pork and beef, suggesting potential for certain microalgae products in the food sector ( Smetana et al., 2017).

In agriculture, de Siqueira Castro et al. (2020) compared microalgae-based biofertilizer to conventional triple superphosphate fertilizer. The study found that the biofertilizer had higher environmental impacts across all categories, particularly in climate change and terrestrial ecotoxicity. The main contributors to these impacts were electricity use for cultivation, harvesting, and drying of the microalgae biomass. An optimized scenario using photovoltaic energy, gravitational biomass settling, and natural drying reduced these impacts significantly ( de Siqueira Castro et al., 2020).

While a substantial body of research exists on the environmental impacts of microalgae-based products, there is a critical need to compare the potential of carbon capture and utilization (CCU) across different applications. This study aims to address this gap by conducting a comprehensive life cycle assessment to compare the environmental performance of biofertilizers, animal feed, and biodiesel from microalgae produced from captured CO _2_. These three products were selected due to their significant market potential and environmental implications. In aquaculture, microalgae species like *Isochrysis, Pavlova, and Chlorella vulgaris* offer superior nutrition and health benefits compared to traditional feed sources ( Dineshbabu et al., 2019). With global fish production projected to reach 209 million tonnes by 2030, microalgae-based feeds are poised to play a crucial role in meeting this growing demand sustainably ( Dineshbabu et al., 2019). In the energy sector, global biodiesel consumption has surged from 2.2 million tonnes in 2004 to 65.86 million tonnes in 2023, with expectations to exceed 75 million tonnes by 2030 ( Statista, 2024). Thailand, in particular, has set an ambitious target of 11 million liters per day of biodiesel production by 2037, up from 4.28 million liters in 2018 ( Intamano et al., 2024). The biofertilizer market is also experiencing significant growth, with projections indicating an increase from USD 1106 million in 2016 to USD 3124 million by 2024, at a CAGR of 14.2% ( Miranda et al., 2024).

In light of these promising market developments, the research will delve into the environmental impacts of two microalgae strains across these distinct applications. The study has two primary objectives. First, this study rigorously evaluates the environmental impacts of three microalgae products (biodiesel, fish feed, and biofertilizer) under three distinct electricity scenarios. These scenarios include Thailand’s current electricity mix as of 2020, a hybrid scenario combining on-site renewable energy production with grid electricity, and a fully renewable energy scenario. The potential CO _2_ emission reductions and their associated benefits are evaluated for the top-performing scenarios. This analysis incorporates a comprehensive assessment of emissions from diverse sources, including combustion of fossil fuels, land-use changes, and potential emissions mitigated through industrial processes such as carbon capture at power plants. Quantitative analysis of CO _2_ utilization pathways will help identify the most eco-friendly and feasible CCU technologies for Thailand. The study’s results will contribute to Thailand’s low-carbon transition and inform policymakers and industry about the most promising CCU strategies.

## 2. Methods

The environmental implications of transitioning from conventional to microalgae-derived products were assessed through a rigorous life cycle assessment framework, adhering to ISO 14040 and 14044 standards ( ISO, 2006a; ISO, 2006b) for methodological consistency.

### 2.1 Goal and scope definition

This study aims to determine which of three microalgae-based products has the least environmental impact. The intended application is to draw comparative conclusions about the environmental impacts of various microalgae products produced from carbon dioxide captured using amine-based technology from a nearby power plant. The impact of transportation is not considered due to the proximity of the capture source. The microalgae products examined in this study—biodiesel, fish feed, and biofertilizer—were selected based on their potential commercial viability and relevance to current market demands. Results are aimed at informing potential stakeholders, including policymakers and industry leaders, about the feasibility and environmental implications of microalgae technology as a use for captured carbon dioxide. The technologies employed in this study, such as carbon capture, cultivation, harvesting, and product conversion processes, are limited to the best available options known at this time. The functional unit is one million tons of utilized captured carbon dioxide. This is measured as the amount of carbon injected into the photobioreactor.

**Figure 1.**
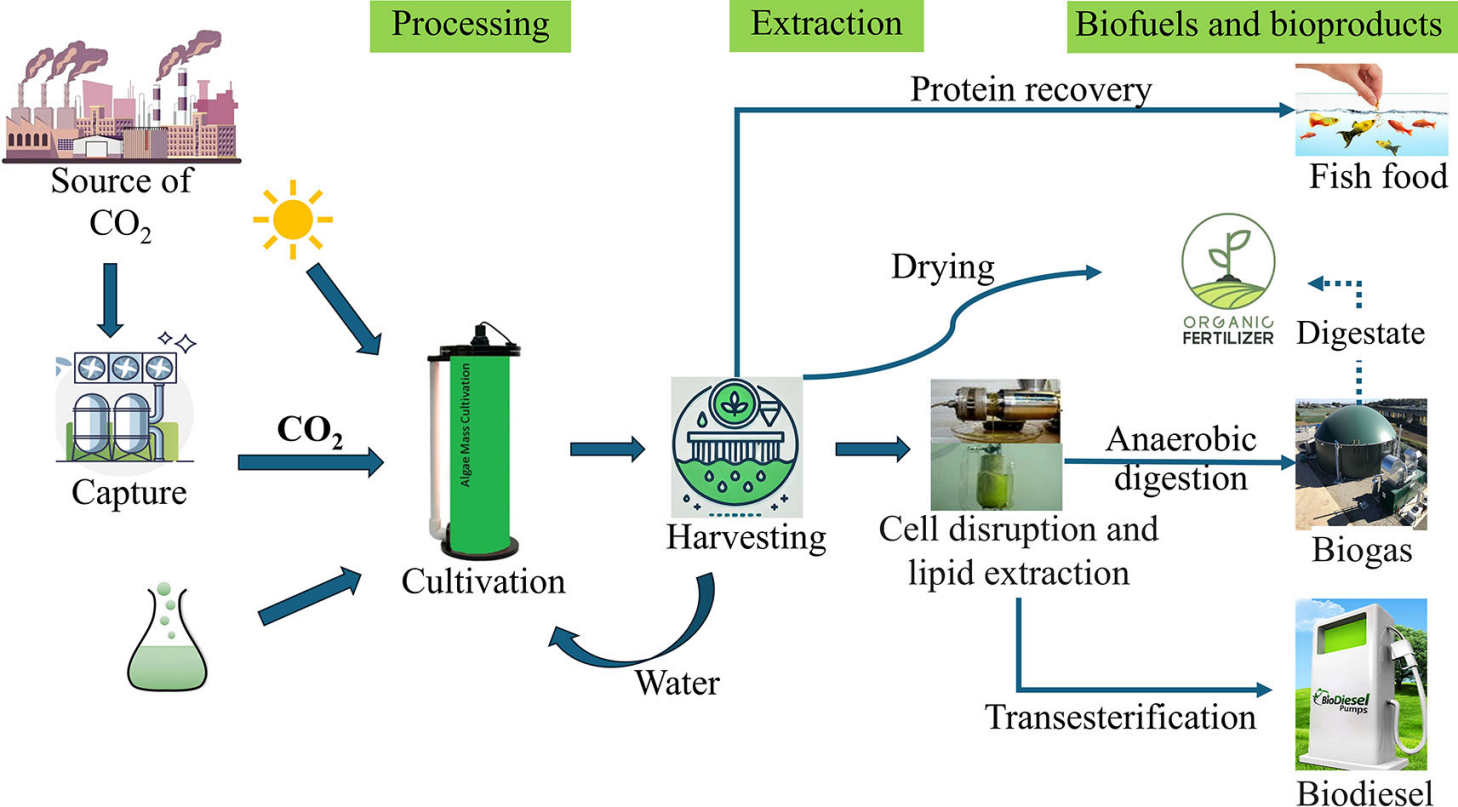
System boundary diagram for biofuels and bioproducts from microalgae.

However, as these technologies are likely to continue developing in the future, potential improvements are considered in this study, in addition to the current state of the art. Initially, the study uses Thailand’s 2020 energy mix as a baseline scenario. In Thailand’s 2020 energy mix (TH20), fossil fuels dominate, with natural gas providing 57% of electricity, coal 19%, and oil 5.8%. Renewable sources contribute modestly, with biofuels at 9.8%, solar PV 2.7%, and wind 1.6%, while hydro accounts for 3.7% and waste for 0.5% of the total generation. To extend the applicability of the results beyond Thailand and explore potential future energy landscapes, the analysis includes two additional energy scenarios: a balanced mix of 50% renewable energy and 50% grid energy (based on the 2020 mix), and a 100% renewable energy scenario. These scenarios were selected based on specific criteria: representation of current conditions (Thailand’s 2020 mix), exploration of a transitional state (50% renewable + 50% grid), and examination of a fully renewable future (100% renewable). Nine scenarios are created by combining each of the three microalgae products with these three energy scenarios. For clarity and brevity, abbreviations are used throughout the study: BF, BD, and FF represent biofertilizer, biodiesel, and fish feed respectively, while the energy scenarios are abbreviated as follows: 100% for 100% Renewable energy, 50% for 50% Renewable energy + 50% Thailand’s electricity mix in 2020, and TH20 for Thailand’s electricity mix in 2020. This approach allows for a comprehensive assessment of how varying levels of renewable energy integration could impact the environmental performance of different microalgae-based carbon capture and utilization technologies. This study has expanded its scope to include a cradle-to-grave analysis, covering carbon capture, microalgae cultivation, production, and use of the three products ( Figure 1). The emissions source was excluded from the system boundary since this study focuses on evaluating and comparing CO _2_-based product production pathways, specifically assessing their suitability for carbon utilization in terms of environmental impact. Substitution was used for the displacement of conventional fossil-based diesel, conventional fish feed, and conventional mineral fertilizer. This allows for comparison based only on the function of utilizing captured carbon and not the individual functions of the three products. The data requirements for this study are the quantitative measures of emissions and resource use.

### 2.2. Life cycle inventory

All data inputs were collected from secondary sources, including life cycle inventory databases and published literature (Table S1) (underlying data). Agri-footprint, Industry Data 2.0, and ecoinvent 3 databases served as the sources for background input data. These inputs include tubular photobioreactor construction, cleaning materials, and system inputs such as nitrogen and phosphorus fertilizers. Carbon capture data was obtained from ( Rafiq et al., 2024a; Win et al., 2023), while cultivation and harvesting data were sourced from ( Bradley et al., 2023; Grierson et al., 2013; Schade and Meier, 2020). Data for subsequent microalgae-based products (biodiesel, fish feed, and biofertilizer) were collected from ( Bradley et al., 2023; Fenton et al., 2014; Grierson et al., 2013; Li et al., 2022; Smetana et al., 2017) and then adapted to the functional unit. Data for the substitution of traditional production of fish feed was sourced from literature as well ( Pelletier and Tyedmers, 2010; Yacout et al., 2016).

*2.2.1 Biodiesel production*

*Nannochloropsis sp.* was selected for this study due to its advantageous characteristics for biodiesel production, particularly its high lipid and protein contents. The nutrient composition of this microalgae species varies significantly across literature, influenced by factors such as nutrient availability and environmental conditions. For this study, a protein content of 30% of dry matter was adopted, aligning with the consensus among several research findings ( Molino et al., 2018; Paes et al., 2016). The lipid content of *Nannochloropsis sp.* exhibits considerable variability, ranging from 5% to 44% of dry matter under optimal growth conditions. Taking into account this variability, an average lipid content of 21% of dry matter was used for this study. Additionally, the eicosapentaenoic acid content, an important omega-3 fatty acid, averages around 4.2% of dry matter ( Molino et al., 2018; Schade and Meier, 2020).

**Figure 2.**
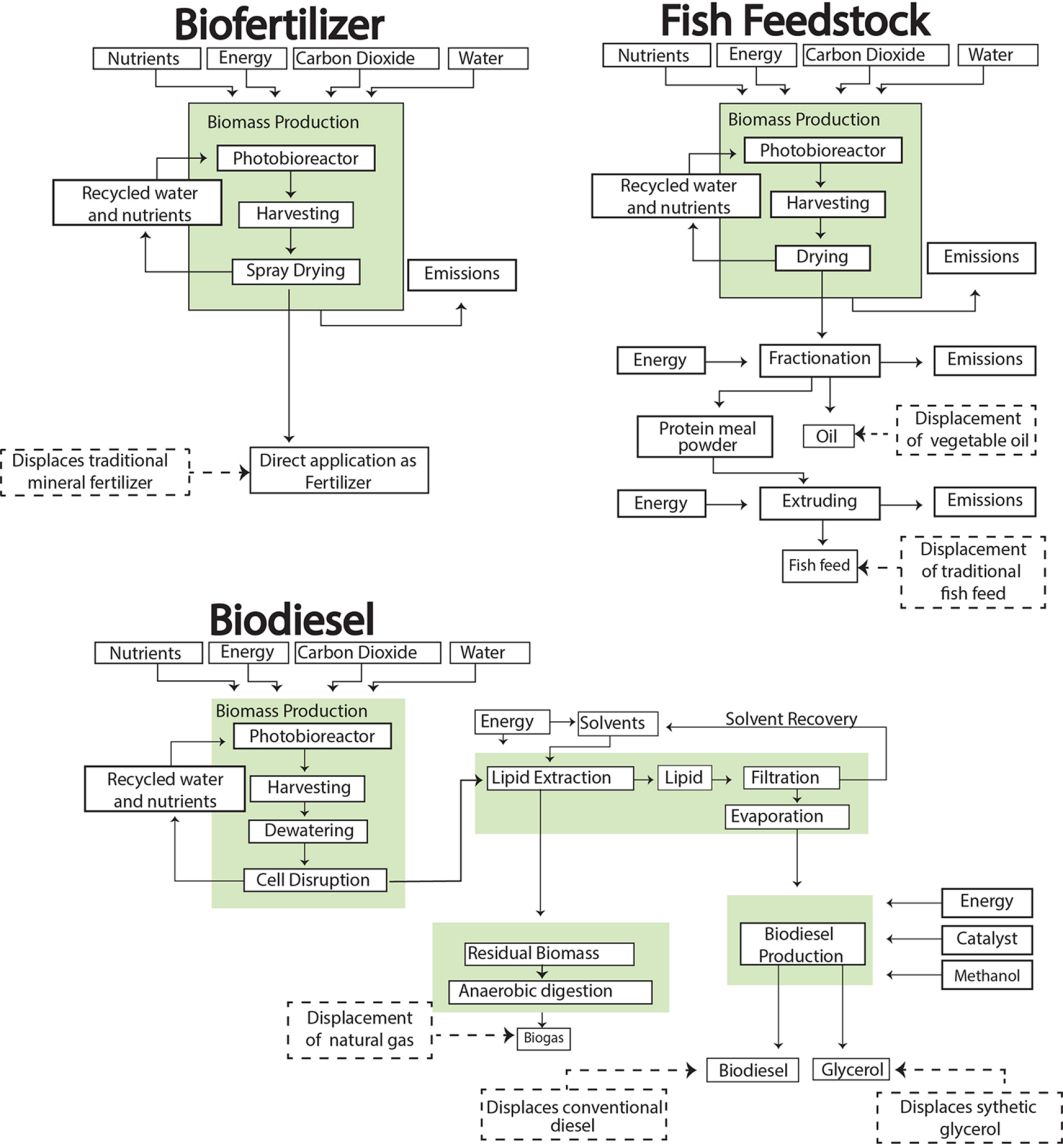
Illustration of the complete cradle-to-grave processes for biofuels and bioproducts production.

In this study, the microalgae are cultivated in borosilicate glass tubular photobioreactors, chosen for their high surface area-to-volume ratio and efficient light utilization [37]. Following cultivation, the biomass undergoes centrifugation for harvesting, followed by lipid extraction ( Figure 2). The extracted lipids are then converted to biodiesel through transesterification, yielding glycerol as a co-product ( Fenton et al., 2014; Schade and Meier, 2020). Substitution was employed for multiple products and co-products throughout the process. The primary output, biodiesel, substitutes conventional diesel fuel on an energy-equivalent basis (37.5 MJ/kg for biodiesel compared to 43 MJ/kg for conventional diesel). Glycerol, a co-product generated at approximately 10% of the biodiesel mass, substitutes synthetic glycerol on a mass-equivalent basis ( Rodrigues et al., 2017). The residual biomass remaining after lipid extraction was utilized for biomethane production through anaerobic digestion. Methane yield from *Nannochloropsis sp.* was approximately 0.35 L per gram of volatile solids, with volatile solids comprising 79% of the total solids in the raw biomass ( Bohutskyi et al., 2014; Torres et al., 2023). The produced biomethane substitutes natural gas, providing a more sustainable alternative to this fossil fuel. The digestate left after biomethane production, rich in nutrients such as nitrogen, phosphorus, and potassium, was used as a biofertilizer. This nutrient-rich digestate further contributed an energy offset of 186.37 MJ/m ^3^ when substituting conventional fertilizers ( Fenton et al., 2014).

*2.2.2 Fish feed and biofertilizer production*

For production of both fish feed and biofertilizer, *Chlorella vulgaris* was assumed as the algal strain. In the case of biofertilizer, chlorella, spirulina, and other algae species have shown benefits to crop growth including increased root and shoot length ( El Arroussi et al., 2018). *Chlorella vulgaris* has also been shown to increase organic matter in soil ( Alvarenga et al., 2023). *Chlorella vulgaris* was selected as it was the strain used to support evidence that microalgae could replace traditional mineral fertilizer ( Schreiber et al., 2018). In the case of fish feed cultivation, *Chlorella vulgaris* was selected as it has a suitable composition of proteins, carbohydrates, and lipids for animal feed. It was found to have less environmental impacts than *A. platensis* ( Smetana et al., 2017). *Chlorella vulgaris* and *A. platensis* are the most widely recognized strains when studying food production ( Smetana et al., 2017). Construction and operation of a tubular photobioreactor would not be affected by the algae strain being grown. Therefore, the process had identical inputs to that of the algae strain selected for biodiesel. The same approach was applied for harvesting and centrifugation. Centrifugation could be employed to separate the dry biomass from the water regardless of strain because it does not rely on specific strain properties.

Once cultivated and harvested, microalgae can be fractionated into a protein powder that can be used for a variety of animal feeds ( Smetana et al., 2017). In this study, fish feed was chosen as the specific feed produced as microalgae are well suited for it. Microalgae has appropriate proportions of proteins, lipids, and carbohydrates for fish meal and already contains essential amino acids required in fish feed, eliminating the need to add them as a secondary step. Microalgae-derived fish feed can be used as a refrigerated paste or as a dry product created by either pelletizing or extruding ( Nagappan et al., 2021). A dry product is in higher demand than refrigerated paste, and the extruder is the most common method for this process ( Nagappan et al., 2021); therefore, this method was chosen for this study.

The fractionation process for *Chlorella* requires 0.7 kWh/kg of energy, yielding 0.85 kg of protein-rich powder and 0.15 kg of oil ( Smetana et al., 2017). The extracted oil substitutes conventional vegetable oil on a mass basis. This substitution accounts for the environmental impacts avoided by not producing an equivalent amount of conventional vegetable oil (soybean oil). The protein powder, containing approximately 45−55% protein, is then mixed with water and subjected to an extrusion process to produce fish feed pellets ( El-Naggar et al., 2020; Smetana et al., 2017). The extrusion process requires an additional 0.13 kWh of electricity per kilogram of feed material processed, according to scaled data from industrial feed production ( Li et al., 2022). The resulting microalgae-based fish feed substitutes conventional fish food on a mass basis ( Yacout et al., 2016), leveraging its high protein content and balanced nutrient profile to meet the dietary needs of farmed fish.

After centrifugation, microalgae are dehydrated for direct application as a biofertilizer. In order to dry the microalgae, both spray drying and lyophilization are available methods ( González et al., 2020). For this study, spray drying was assumed as the drying method, and energy inputs were assumed as 4 GJ/tonne resulting in an 80 percent yield ( Grierson et al., 2013). It was also assumed that microalgae are able to directly replace mineral fertilizer in the form of 15:15:15 NPK (nitrogen, phosphorus, potassium). Studies have shown that microalgae in a ratio of two percent algae to 98 percent soil met the requirements for use as a solid bio-based fertilizer. The microalgae applied as fertilizer demonstrated significant plant growth compared to ammonium sulfate when applied to both ryegrass and barley ( González et al., 2020). Additionally, it has been demonstrated that in nutrient-deficient soils, algae resulted in comparable plant growth to plants treated with mineral fertilizer ( Schreiber et al., 2018).

The fertilizers were compared based on the amount of nitrogen applied to the soil to credit traditional fertilizer production. They cannot be compared based on volume as microalgae-based fertilizer has a smaller amount of plant nutrients per kilogram. Inputs into microalgae fertilizer production indicated 41 kilograms of nitrogen per tonne of CO _2_ utilized. The selected mineral fertilizer (NPK 15:15:15) is fifteen percent nitrogen. Therefore, to be equivalent to the 555 kg of dry biomass resulting from 1 tonne of carbon dioxide, the production of 273 kg of mineral fertilizer is displaced.

### 2.3 Life cycle impact assessment

The study utilized SimaPro 9.5, a specialized LCA software ( PRé, 2023), along with the ReCiPe 2016 LCIA method ( Huijbregts et al. 2016), to analyze the environmental performance of the three products. The analysis focused on five key impact categories, including a combined midpoint category for global warming (encompassing impacts on both human health and terrestrial ecosystems), along with three endpoint categories: human health (measured in disability-adjusted life years, DALY), ecosystem quality (represented as the Potentially Disappeared Fraction of species per area-year, species.yr), and resource scarcity (expressed in USD2013). These midpoint impact categories were chosen for their relevance in assessing the effectiveness of CCU technologies, which aim to capture and utilize CO _2_, thereby potentially reducing global warming impacts. Including endpoint impact categories was essential for gaining a comprehensive understanding of the final environmental damage translating specific environmental pressures into broader areas of protection ( Huijbregts et al. 2016).

### 2.4 Cradle-to-grave CO _2_ emissions reduction

**Figure 3.**
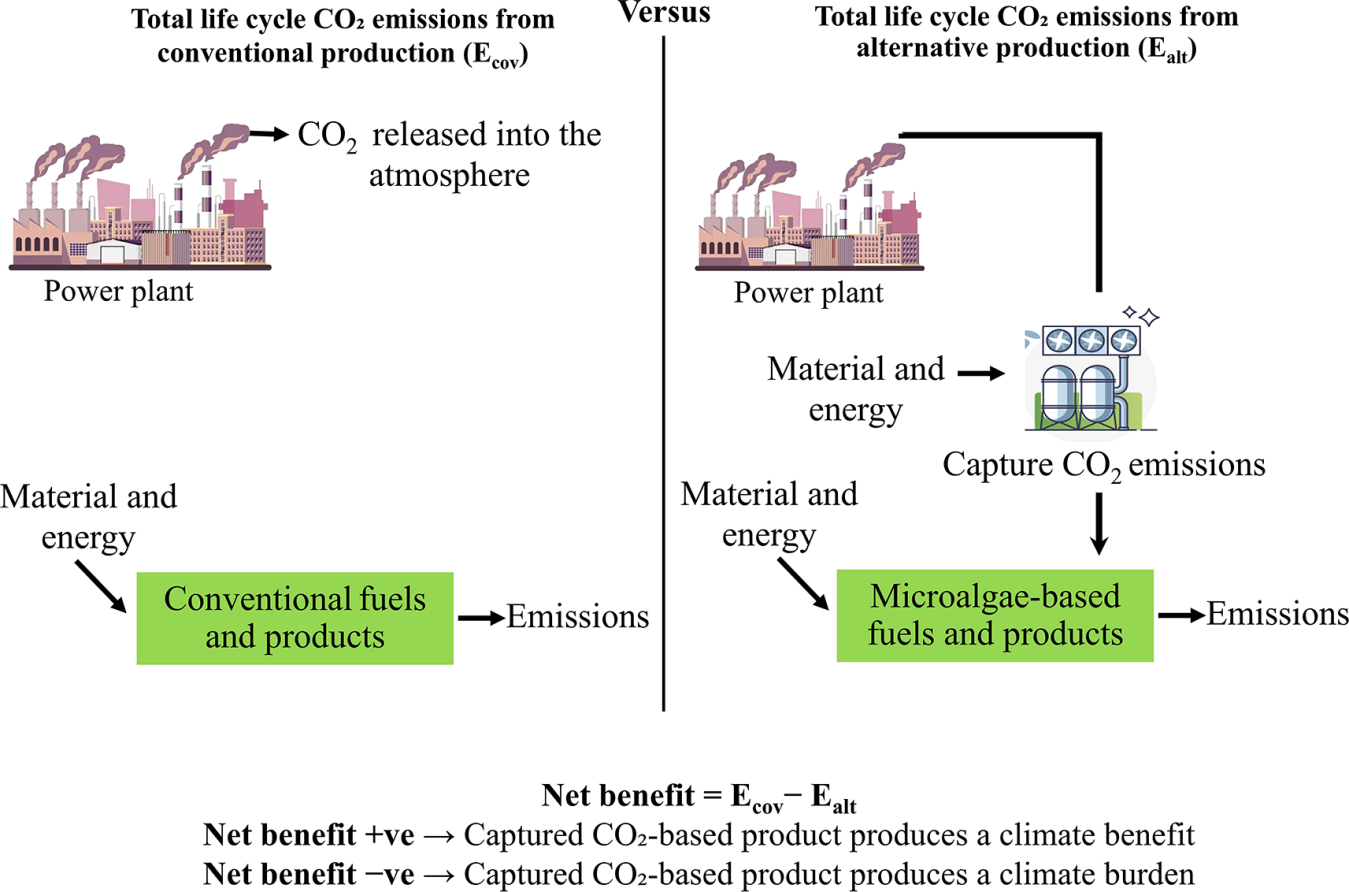
CO _2_ emissions comparison for traditional and CO _2_-utilizing bioproducts manufacturing.

To assess the overall advantage of reducing carbon footprint in the optimal scenario, this study covers CO _2_ emissions from various sources, including fossil fuel use and land-use changes, as well as emissions prevented in the production processes of the best-case scenarios. Fossil-based emissions are thoroughly assessed, covering the entire process from carbon capture to the production of algae-based products. This evaluation also accounts for the avoided emissions that result from replacing conventional fossil-based products with CO _2_-utilizing alternatives. The approach employs a comparative evaluation of life cycle CO _2_ emissions between traditional method and alternative CO _2_-based pathways as shown in Figure 3. This comparison quantifies the net climate effect, highlighting the reduction in total CO _2_ emissions achieved through the alternative production pathway.

## 3. Results and discussion

**Figure 4.**
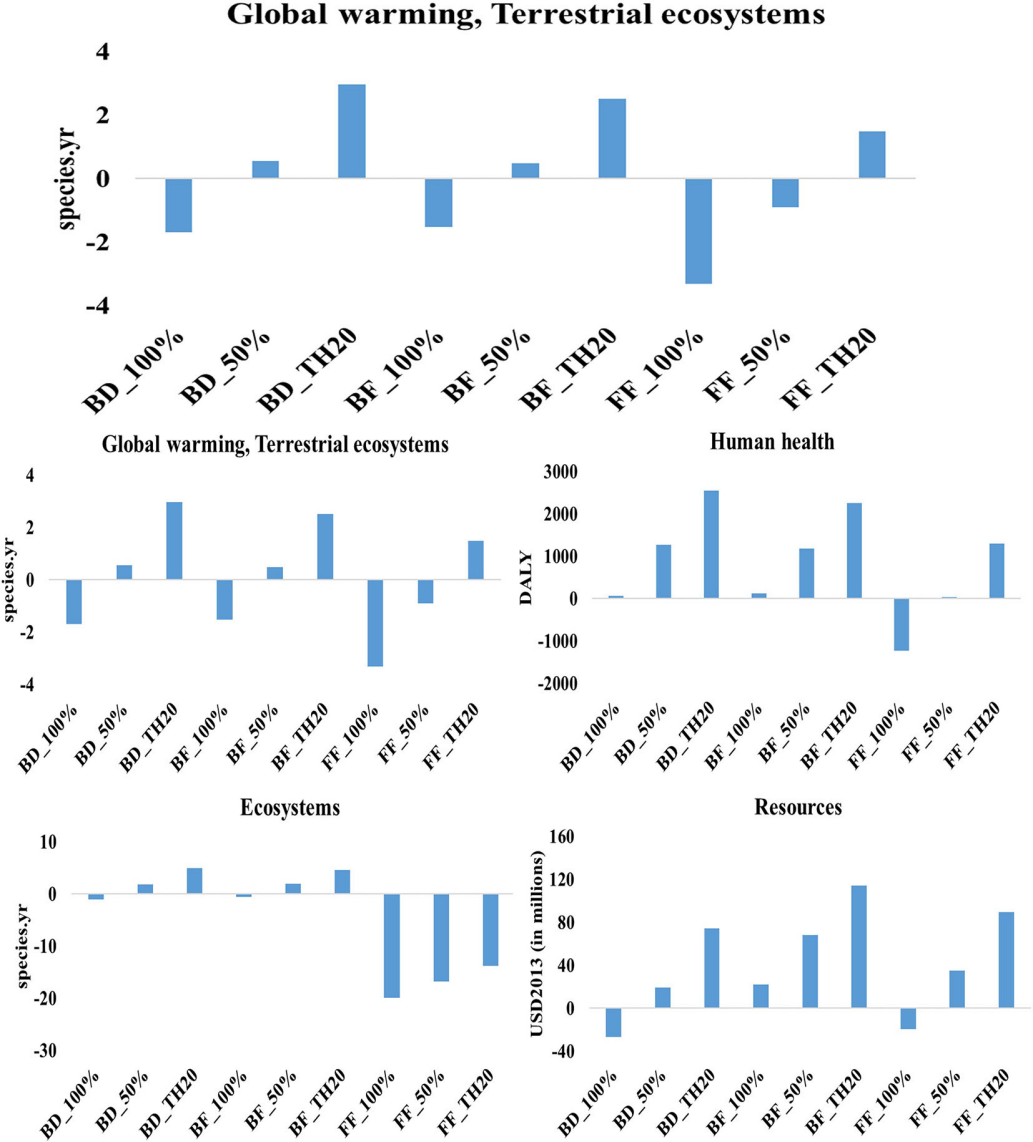
Environmental impacts of microalgae-based biodiesel, biofertilizer, and fish feed across varied electricity scenarios.

The life cycle assessment of microalgae-derived biodiesel, fish feed, and biofertilizer highlights their substantial potential to support sustainable development and contribute to climate change mitigation. This study evaluated nine scenarios, each considering different electricity profiles: current Thai grid mix, 50% renewable energy, and 100% renewable energy. The scenarios were analyzed using the ReCiPe 2016 method across multiple environmental impact categories. The findings indicate that the environmental performance of each product is significantly influenced by the energy sources used during production. The results indicate that transitioning from a fossil-fuel-dominated grid to 100% renewable electricity leads to substantial reductions in environmental impacts across all microalgae-based products ( Figure 4). Among the three products, fish feed produced with 100% renewable energy achieves the most significant reductions in global warming impacts, with −1095 DALY (human health) and −3 species.yr (terrestrial ecosystems) per million tonne CO _2_ utilization (functional unit), demonstrating its strong potential as a low-carbon product. Similarly, biodiesel under fully renewable conditions achieves notable reductions, with −556 DALY (human health) and −2 species.yr (terrestrial ecosystems), underscoring its role as a cleaner alternative to conventional fuels. Biofertilizer, while also benefiting from renewable energy integration, shows lower reductions, recording −503 DALY and −1.5 species.yr, indicating its potential to reduce emissions, albeit to a lesser extent compared to fish feed and biodiesel.

The impact on human health and ecosystems further underscores the benefits of renewable energy adoption. Fish feed produced with 100% renewable energy achieves the highest reduction in human health impacts, with −1230 DALY, illustrating its capacity to minimize health-related risks linked to the production process. Biodiesel produced with 100% renewable energy results in 68 DALY, highlighting challenges in reducing health-related impacts compared to fish feed. In terms of ecosystem quality, fish feed with 100% renewable energy achieves a decrease of −20 species.yr, outperforming biodiesel and biofertilizer. However, despite achieving notable reductions in global warming and ecosystem impacts, biofertilizer under 100% renewable conditions records an increase of 118 DALY in human health impacts, suggesting that further process optimization is needed to enhance its overall environmental sustainability. The adoption of 100% renewable energy significantly reduces resource-related impacts across all products. Biodiesel shows substantial progress, with a reduction of 26 million USD2013, underscoring its enhanced resource efficiency under a fully renewable energy mix. Following closely, fish feed demonstrates the lowest resource impact, achieving a reduction of 20 million USD2013, indicating significant improvements in resource efficiency. In contrast, biofertilizer under 100% renewable conditions registers an impact of 23 million USD2013, highlighting a need for better resource management strategies to achieve more sustainable outcomes.

Scenarios using 50% renewable energy mix exhibit moderate reductions compared to fully renewable scenarios. Fish feed and biodiesel produced under this energy mix show reduced impacts on global warming and human health, though these reductions are less pronounced than those observed under a 100% renewable energy mix. For example, fish feed produced with 50% renewable energy achieves a reduction of −302 DALY in global warming impacts on human health, while biodiesel records 186 DALY, reflecting improved but still comparatively significant impacts. This suggests that partial integration of renewable energy can enhance sustainability, but it does not provide the same benefits as a fully renewable approach. The highest environmental impacts are seen in scenarios that rely on Thailand’s current electricity grid mix, which is heavily dependent on fossil fuels. In these scenarios, fish feed production results in 491 DALY for global warming impacts on human health and 1.5 species.yr for terrestrial ecosystem impacts, indicating substantial environmental burdens. Similarly, biofertilizer and biodiesel produced under the current grid mix exhibit high impacts across global warming, human health, and resource scarcity categories. For instance, biofertilizer records 1182 DALY for human health impacts, while biodiesel registers 1273 DALY, making these the least sustainable scenarios due to their reliance on fossil-based electricity.

Carbon capture contributes significantly to the environmental performance of microalgae-based products. For the functional unit of capturing one million tonnes of CO _2_ using amine-based technology, substantial benefits are observed across impact categories ( Table 1). Under the 2020 grid mix, the global warming impact on human health is reduced by 719 DALY, which further improves to 829 DALY when shifting to a 100% renewable energy scenario, reflecting a 15% enhancement. Similar improvements are noted in global warming impacts on terrestrial ecosystems, becoming more pronounced with increased renewable energy integration. The most significant improvement is seen in resource scarcity, with a 42% reduction in economic impacts achieved when using a fully renewable energy mix. Additionally, human health and ecosystem quality benefits range from 21% to 30%, indicating moderate but meaningful reductions across these categories. This analysis demonstrates that carbon capture, especially when combined with renewable energy sources, can play a pivotal role in enhancing the sustainability of microalgae-based products.

**Table 1.** Environmental impacts of implementing carbon capture in microalgae-based production across varying electricity scenarios.

Impact category	Unit	Current Grid Mix	50% Renewable	100% Renewable
Global warming, Human health	DALY	-718.54	-773.59	-828.64
Global warming, Terrestrial ecosystems	species.yr	-2.17	-2.33	-2.5
Human health	DALY	-581.88	-669.77	-757.66
Ecosystems	species.yr	-1.98	-2.19	-2.4
Resources	USD2013 (millions)	18.21	14.43	10.64

### 3.1 Contribution analysis

**Figure 5.**
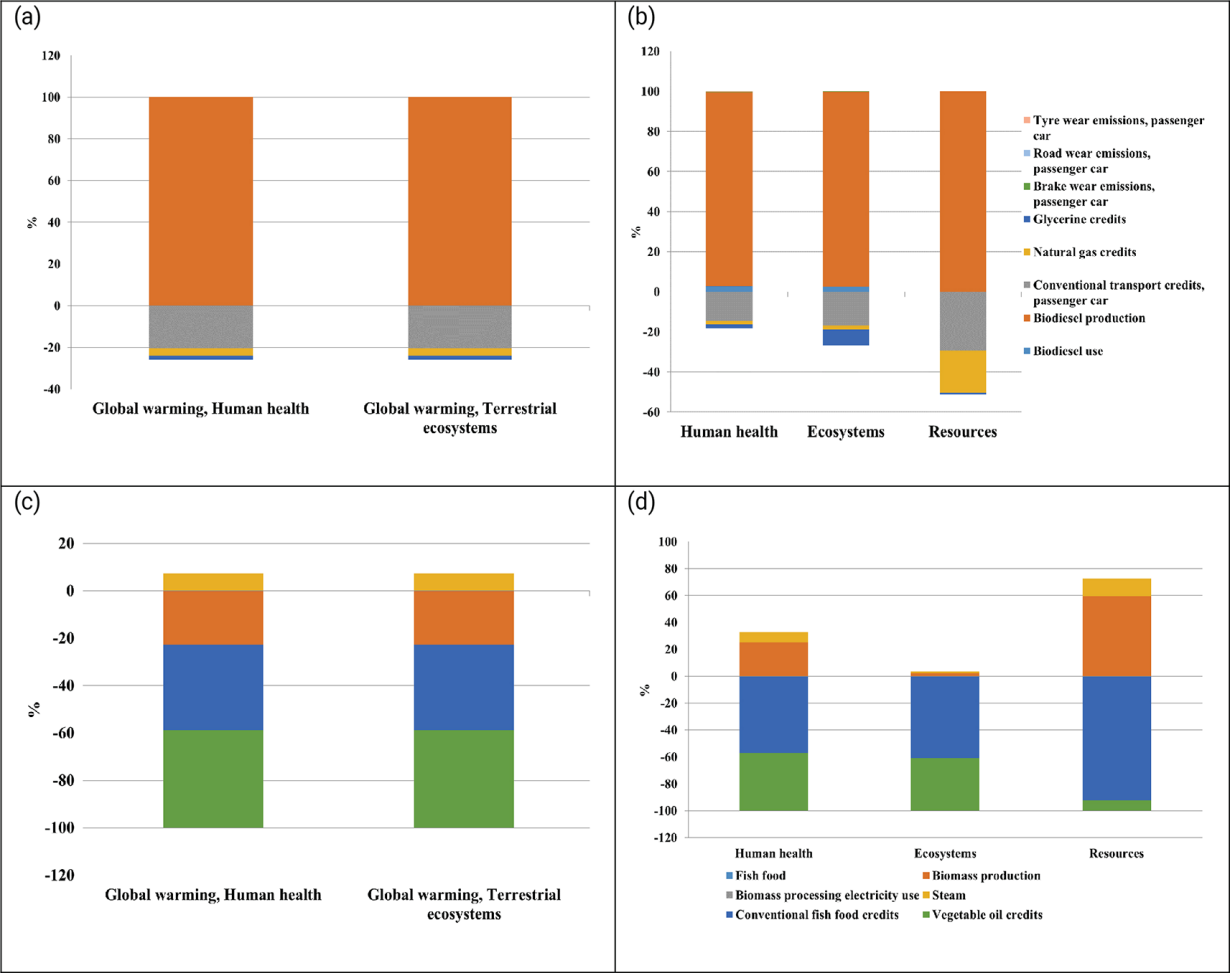
Comparative assessment of extremal scenarios in microalgal production systems. Environmental impact analysis contrasting: (a) Midpoint impact evaluation for biodiesel production under suboptimal conditions utilizing the current grid electricity mix; (b) Associated endpoint impact indicators for the biodiesel worst-case scenario; (c) Midpoint impact categories for fish feed production under optimal conditions with 100% renewable energy integration; and (d) Corresponding endpoint impact metrics for the optimized fish feed scenario. This multi-panel analysis illustrates the spectrum of environmental implications across different production pathways and energy source configurations.

The contribution analysis of microalgae-based biodiesel production under worst-case scenario highlights that biodiesel production is the primary driver of environmental impacts across all categories. Substitution credits from conventional transport, natural gas, and glycerine significantly reduce the global warming impacts on human health and terrestrial ecosystems by approximately 21%, 3%, and 2%, respectively ( Figure 5). Additionally, glycerine credits reduce impacts in the ecosystem category by about 8%. In resource use, substitution credits from conventional transport and natural gas provide notable reductions of approximately 29% and 21%, respectively. While these credits offer significant reductions, they are insufficient to fully counter the high emissions from the current fossil-based electricity mix. This analysis underscores the need for cleaner energy solutions in biomass production processes to achieve more effective environmental improvements.

Conversely, fish feed production under best-case conditions with 100% renewable energy shows significant improvements in environmental performance. Biomass production achieves a 22% reduction in global warming impacts, further enhanced by substitution credits from conventional fish feed (36%) and vegetable oil replacement (41%). Although steam use causes a minor 7% increase, the overall impact on the environment still remains positive. While biomass production increases human health impacts by 25%, substantial credits from conventional feed (−57%) and vegetable oil (−43%) lead to net reductions. Ecosystem and resource impact categories exhibit similar positive trends, driven by effective substitutions. These findings align with previous studies that identify two major challenges in algae-based systems. The first is the intensive energy demand for processes like pumping, mixing, drying, and lipid extraction, especially when reliant on fossil fuels. The second challenge is nutrient management, particularly nitrogen and phosphorus usage, where inefficiencies can increase eutrophication and greenhouse gas emissions ( Bradley et al., 2023; Fenton et al., 2014; Schade and Meier, 2020).

Despite these challenges, the present study also found that *Chlorella vulgaris* has the potential to replace high-material and energy-intensive aquaculture feeds. Its viability as an alternative feed is supported by its favorable nutritional profile: 45% protein, 20% fat, 20% carbohydrates, 10% minerals and vitamins, and 5% fiber (w/w, dry basis) ( El-Naggar et al., 2020). Modelling the substitution of conventional feed with *Chlorella vulgaris* reveals even greater environmental benefits than previously anticipated. Table 2 compares microalgae-based feed (FF_TH20) with conventional aquaculture feed production. The results show significant reductions in global warming potential for both human health and terrestrial ecosystems. The substitution substantially reduces human health and ecosystem impacts, while resource impacts decrease only marginally. These findings demonstrate *Chlorella vulgaris’* potential as a sustainable aquaculture feed alternative, though resource efficiency needs optimization. To make the process even more efficient, implementing emerging approaches like bioflocculation, spontaneous flocculation, and attached growth systems show promise for more efficient and economical harvesting, potentially reducing chemical use and improving harvested biomass concentration ( Bilad et al., 2012; de Assis et al., 2019; de Assis et al., 2017; Eliseus et al., 2017). However, the drying step remains a challenge, with various options like solar, drum, spray, and freeze-drying each presenting trade-offs between energy use, cost, and product quality ( Milledge and Heaven, 2013). These strategies, combined with ongoing research into energy-efficient and environmentally friendly methods, aim to optimize both energy use and nutrient management in algal biofuel production, addressing the key challenges identified in life cycle assessments and paving the way for more sustainable algae-based systems in the future. Overall, the potential for algae-based systems to contribute to environmental sustainability is significant, as evidenced by various studies showing substantial reductions in environmental impacts when transitioning to renewable energy sources. This highlights the importance of not only increasing the share of renewable energy in the grid mix but also improving the energy efficiency of production processes themselves. With continued advancements and a focus on overcoming key challenges, algae-based CCU and bio-product production hold promise for playing a pivotal role in mitigating climate change and promoting a more sustainable and resilient future, a vision that is thoroughly supported by the results and conclusions of the current study.

**Table 2.** Comparative environmental impacts per million tonne of microalgae-based (FF_TH20) and conventional fish feed.

Impact category	Unit	Microalgae-based fish feed	Conventional fish feed
Global warming, Human health	DALY	1044	3726
Global warming, Terrestrial ecosystems	species.yr	3	11
Human health	DALY	2770	6159
Ecosystems	species.yr	-29	34
Resources	USD2013 (million)	190	193

### 3.2 Application of top-performing scenarios

Microalgae CCU provides biomass-containing compounds useful for pharmaceuticals, food, and biofuels ( Zhao and Su, 2020). However, realizing their full potential requires overcoming key challenges identified through life cycle assessments. The optimization of microalgae CCU systems depends on two critical factors—the refinement of system processes for greater efficiency and the careful integration of renewable energy sources, both of which are crucial for improving environmental performance, as examined below.

*3.2.1 Impact of system efficiency and renewable energy*

For carbon capture, Win et al. (2023) reported that capturing one tonne of CO _2_ requires approximately 378 kWh of energy, highlighting the significant energy input needed for carbon capture systems. This energy requirement is closely tied to the efficiency of the CO _2_ utilization process, which varies significantly across various studies, ranging from 40% to 93.7%, depending on specific conditions and technologies employed for microalgae cultivation ( Ighalo et al., 2022). The results discussed in this study are based on a carbon utilization efficiency of 75%, which falls within the range reported in the literature. This relatively high efficiency suggests potentially lower energy requirements and environmental impacts compared to systems with lower efficiencies, as the energy needed per unit of CO _2_ captured would be reduced.

**Figure 6.**
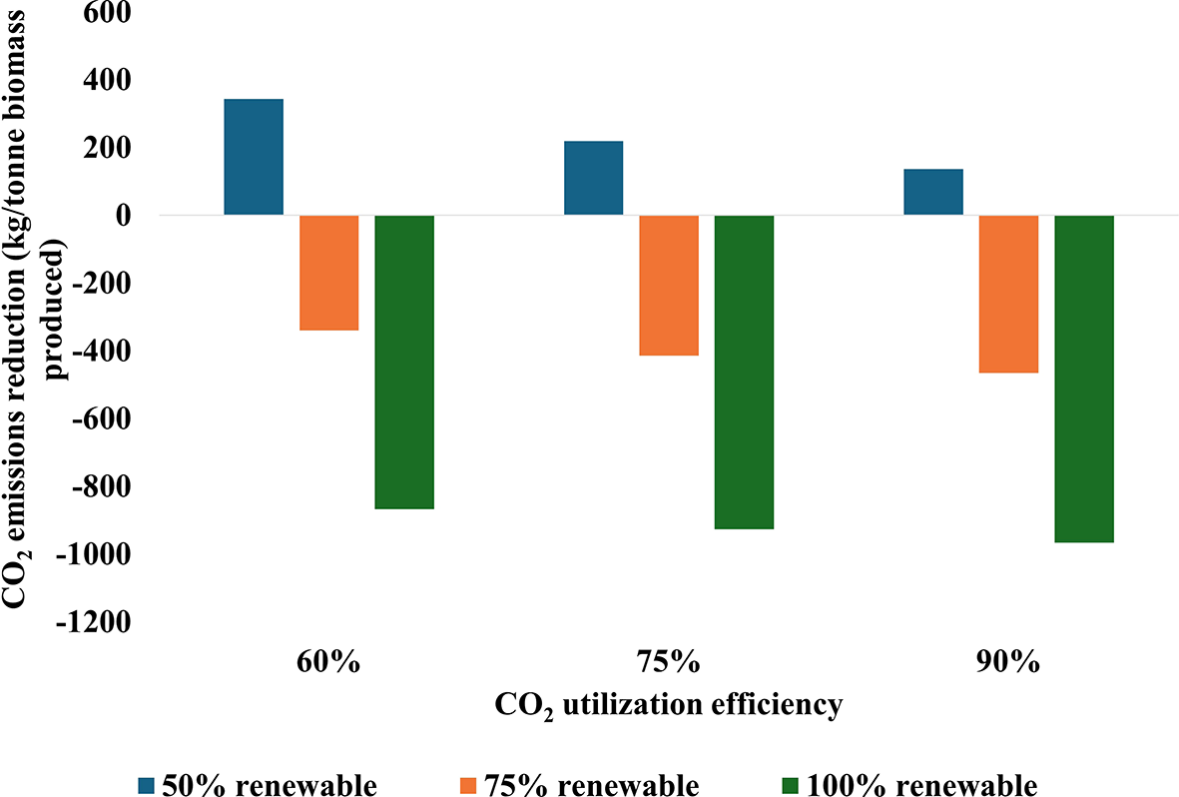
CO _2_ emissions reduction during biomass production as influenced by utilization efficiency and renewable energy integration.

Figure 6 illustrates the impact of CO _2_ utilization efficiency and renewable energy integration on CO _2_ emissions reduction in cultivation systems. The data reveal a clear trend: increasing CO _2_ utilization efficiency from 60% to 90% and renewable energy integration from 50% to 100% significantly improve CO _2_ reduction rates. Notably, with 100% renewable energy and 75% CO _2_ utilization efficiency, the reduction rate nearly matches that of the scenario with 90% CO _2_ utilization efficiency and 100% renewable energy. Importantly, in both the 75% and 100% renewable energy scenarios, all efficiency levels result in a net reduction of CO _2_, highlighting the critical role of renewable energy in achieving positive environmental outcomes. While the combination of high efficiency at 90% and full renewable energy at 100% maximizes CO _2_ reduction, the analysis indicates that balanced improvements in both parameters can yield substantial benefits without necessarily reaching the highest levels in each category. These relationships underscore the importance of optimizing both CO _2_ utilization efficiency and renewable energy integration within carbon capture systems.

The analysis demonstrates a broad range of CO _2_ reduction potential across the scenarios. At the lower end, with 60% efficiency and 50% renewable energy, the system results in net emissions of 343 kg CO _2_ per tonne processed. In contrast, the most favorable scenario with 90% efficiency and 100% renewable energy achieves a reduction of 965 kg CO _2_ per tonne CO _2_ utilized, representing a significant enhancement in carbon utilization performance. Moreover, the energy intensity of CO _2_ utilization could be further reduced by using flue gas directly instead of post-combustion CO _2_ capture. However, direct use of flue gas can affect algae growth and lipid production due to contaminants, with impacts varying by species. Napan et al. (2015) found that at baseline heavy metal concentrations representative of coal-fired flue gas, there was a 12% increase in biomass production and 61% increase in lipid yield for *Scenedesmus obliquus* compared to the control. However, at higher heavy metal concentrations, both growth and lipid production were inhibited ( Hess et al., 2017).

Similarly, Hess et al. (2017) observed that for *Nannochloropsis salina*, the addition of 14 inorganic contaminants at concentrations representative of coal-fired flue gas resulted in a 67% reduction in biomass productivity and a 32% decrease in lipid content compared to the control ( Napan et al., 2015). Despite these challenges, using captured flue gas CO _2_ is still more energy-efficient than virgin commercial CO _2_ ( Clarens et al., 2011). Overall, the CO _2_ source significantly influences the energy requirements and sustainability of algae-based biofuel production. However, careful examination of flue gas composition and its suitability for specific algal strains is crucial for informed decision-making in algae-based biofuel production systems.

*3.2.2 Benefits of carbon capture and utilization*

In developing countries, where fossil fuels often dominate the energy landscape, coal and natural gas power plants continue to be significant sources of CO _2_ emissions. However, these emissions can be repurposed through innovative carbon capture and utilization technologies, offering a pathway to more sustainable practices. One promising application is the cultivation of *Chlorella vulgaris*, a microalga species, to produce fish feed. *Chlorella vulgaris* has shown remarkable potential as an alternative to conventional, energy-intensive aquaculture feeds ( Dineshbabu et al., 2019; Smetana et al., 2017). By utilizing CO _2_ emissions from power plants, the production of this algae-based fish feed can significantly reduce the carbon footprint of both energy and aquaculture sectors. The process requires approximately 0.9 million tonnes of CO _2_ annually to meet Thailand’s fish feed demand of 400,000 tonnes ( Fachrudin, 2024), with each kilogram of algae-based feed produced potentially offsetting around 2.66 kilograms of CO _2_ emissions (Table S1) (underlying data). This estimate accounts for direct emissions from algae cultivation and processing, indirect emissions from land transformation, CO _2_ captured from power plants, and emissions avoided by replacing conventional feed production methods (Table S1) (underlying data). At a market price of USD 1,400 per tonne ( Indiamart, 2024), this industry could generate an annual revenue of USD 560 million. Simultaneously, it would avoid 1.1 million tonnes of CO _2_ emissions per year compared to conventional feed production. This initiative could lead to employment opportunities in carbon capture and utilization industries, as well as in algae cultivation and associated fields, providing an economic boost to the region. Beyond its economic potential, fish feed derived from *Chlorella vulgaris* offers several environmental benefits. It can help reduce pressure on wild fish stocks traditionally used for fishmeal production ( Sheikhzadeh et al., 2024), contributing to marine ecosystem conservation. Additionally, as a protein-rich and nutrient-dense feed, it can improve the efficiency and sustainability of aquaculture operations.

In addition to fish feed production, microalgae cultivation holds great promise for carbon capture and utilization, with applications extending to the production of biodiesel and biofertilizer. The biodiesel production process requires approximately 37.5 million tonnes of CO _2_ annually to meet Thailand’s anticipated biodiesel demand of 4,015 million liters by 2037 as per government plans ( Intamano et al., 2024). A market price of USD 1,000 per tonne ( Data, 2024) would generate approximately USD 3.5 billion in revenue. Importantly, each kilogram of microalgae-based biodiesel avoids approximately 8.5 kg of CO _2_ (see Table S1) (undelrying data) emissions compared to conventional fossil-based diesel, potentially reducing emissions by 30 million tonnes of CO _2_ annually.

Moreover, the conversion of algal biomass into biofertilizer presents a promising market opportunity, requiring about 9 million tonnes of CO _2_ annually to meet the biofertilizer demand of 5 million tonnes ( Post, 2022). Assuming a market price of approximately USD 400 per tonne ( Polene, 2024), this sector could generate substantial revenue of about USD 2 billion each year. Moreover, biofertilizer derived from microalgae offers significant environmental benefits, as each kilogram of this product can avoid approximately 1.2 kg of CO _2_ emissions. This translates to a total reduction of around 6 million tonnes of CO _2_ emissions annually, contributing to climate change mitigation efforts. In 2022, Thailand’s national CO _2_ emissions reached approximately 270 million tonnes ( Ritchie and Roser, 2022), with the power generation sector accounting for about 90 million tonnes ( Statista, 2023), primarily from coal and natural gas. As part of its commitment to combating climate change, Thailand’s Nationally Determined Contribution (NDC) targets a 20-30% reduction in greenhouse gas emissions by 2030 ( Sugsaisakon and Kittipongvises, 2024). The CO _2_ utilization potential through these three microalgae-based products amounts to 47 million tonnes annually, representing 53% of the power sector’s emissions. These products could collectively offset 37 million tonnes of CO _2_ per year, contributing approximately 14% towards Thailand’s total emissions reduction. By leveraging microalgae technology, Thailand could make significant strides in reducing its carbon footprint while fostering sustainable industrial practices.

However, scaling up the production of microalgae using power plant emissions faces several challenges. Substantial investments in infrastructure are required, including CO _2_ capture facilities at power plants, large-scale algae cultivation systems, and processing plants for the final products ( Hepburn et al., 2019). While the cultivation of algae using industrial CO _2_ has been demonstrated on smaller scales, expanding to fully utilize emissions from large power plants necessitates further technological advancements and process optimizations. Market development presents another crucial consideration. Establishing and expanding markets for algae-based products may require efforts in consumer education, quality assurance, and potentially supportive policies to encourage adoption. A favorable regulatory environment, possibly including carbon pricing mechanisms or incentives for CCU technologies, would be instrumental in making these projects economically viable ( Hepburn et al., 2019).

Developing efficient cultivation methods and selecting appropriate locations for production facilities will be key to addressing these concerns. Beyond these implementation challenges, the production of value-added products such as fish feed, biodiesel, and biofertilizer from microalgae using power plant emissions represents a promising approach to emissions reduction and sustainable development. As countries work towards cleaner energy futures, these technologies could serve as valuable transitional solutions, mitigating emissions from existing power infrastructure while supporting food security, energy independence, and agricultural sustainability. The potential for significant CO _2_ reduction, coupled with economic benefits across multiple sectors, makes this an attractive option for countries seeking to balance development needs with climate change mitigation goals. By simultaneously addressing issues in aquaculture, transportation, and agriculture, microalgae-based CCU demonstrates a climate-friendly solution for multiple sectors in the economy. The versatility of these applications offers a pathway to create a circular economy, where waste CO _2_ becomes a valuable resource for multiple industries.

## 4. Conclusions

This life cycle assessment of microalgae-based biodiesel, fish feed, and biofertilizer production in Thailand reveals the significant potential of these products to contribute to sustainable development and climate change mitigation. Among the evaluated products, microalgae-based fish feed demonstrated the most favorable environmental profile across multiple impact categories, particularly under increased renewable energy scenarios. The transition from the current energy mix to higher renewable energy penetration resulted in notable environmental improvements for all three products. Biodiesel production, while showing improvements under increased renewable scenarios, consistently exhibited higher environmental impacts compared to fish feed and biofertilizer. This suggests that prioritizing microalgae cultivation for high-value products like fish feed may offer greater environmental benefits in the near term.

The study highlighted the critical role of both CO _2_ utilization efficiency and renewable energy integration in determining the environmental performance of these systems. With carbon capture energy requirements of approximately 378 kWh per tonne of CO _2_, the efficiency of utilization becomes crucial. The analysis, based on a 75% carbon utilization efficiency, showed that increasing both CO _2_ utilization efficiency and renewable energy integration leads to significant improvements in CO _2_ reduction rates. Notably, scenarios with high renewable energy integration (75-100%) resulted in net CO _2_ reductions across all efficiency levels, underscoring the importance of renewable energy in achieving positive environmental outcomes.

In Thailand, transitioning to microalgae-based alternatives could have a transformative impact across various sectors. Meeting the current annual fish feed demand of 400,000 tonnes with microalgae products could generate approximately USD 560 million in revenue while avoiding 1.1 million tonnes of CO _2_ emissions compared to conventional feed. Fulfilling the anticipated annual biodiesel demand of 4,015 million liters through microalgae production could yield around USD 3.5 billion in revenue and reduce CO _2_ emissions by 30 million tonnes relative to conventional diesel. Additionally, the estimated annual demand of 5 million tonnes for biofertilizer derived from algal biomass could generate about USD 2 billion in revenue each year while potentially reducing CO _2_ emissions by 6 million tonnes annually. Together, these products could offset 37 million tonnes of CO _2_, representing approximately 14% of Thailand’s total emissions. If implemented, this approach could significantly contribute to advancing the country’s NDC goal of a 20-30% reduction in greenhouse gases. This study offers valuable insights for decision-makers, industry stakeholders, and researchers, supporting Thailand’s transition toward more sustainable practices in aquaculture, energy, and agriculture while accelerating its progress toward climate targets.

## Ethical considerations

Ethical review and consent did not apply to this study.

## Reporting guidelines

Reporting guidelines were not applicable to this study.

## Author roles

Rafiq A: Conceptualization, Data Curation, Formal Analysis, Investigation, Methodology, Writing – Original Draft Preparation, Writing – Review & Editing; Morris C: Conceptualization, Data Curation, Investigation, Methodology, Writing – Original Draft Preparation, Writing – Review & Editing; Schudel A: Conceptualization, Data Curation, Investigation, Methodology, Writing – Original Draft Preparation, Writing – Review & Editing; Gheewala SH: Conceptualization, Investigation, Methodology, Project Administration, Resources, Supervision, Validation, Writing – Review & Editing.

## Data Availability

The data underlying this article are available in Figshare: Life Cycle Assessment of microalgae-based products for carbon dioxide utilization in Thailand: biofertilizer, fish feed, and biodiesel. https://doi.org/10.6084/m9.figshare.27715455 ( Rafiq et al., 2024b). This project contains the following underlying data:
•
Table S1. xlsx (Life Cycle Assessment inventory data, calculations for avoided CO _2_ emissions, and data related to environmental impacts) Full credit is given to all co-authors who contributed to generating this dataset. Data are available under the terms of the Creative Commons CC0 license.
