# Supplementary material for: Life Cycle Assessment of Microalgae-Based Products for Carbon Dioxide Utilization in Thailand: Biofertilizer, Fish Feed, and Biodiesel
Source: F1000Res. 2025 Apr 28;13:1503. Originally published 2024 Dec 9. [Version 3] doi: 10.12688/f1000research.159019.3 (PMC12498316; doi:10.12688/f1000research.159019.3)
Supplement: Peer review history

# Reviewer report: Reviewer response for version 3

**DOI:** 10.5256/f1000research.180527.r381348

**Published:** 2025-04-30

**Authors:** Marcin Dębowski

**Affiliations:** 1 University of Warmia and Mazury, Olsztyn, Poland

In my opinion, the manuscript has been adequately improved and is ready for indexing in its present form.

Is the work clearly and accurately presented and does it cite the current literature?

Partly

If applicable, is the statistical analysis and its interpretation appropriate?

Not applicable

Are all the source data underlying the results available to ensure full reproducibility?

Partly

Is the study design appropriate and is the work technically sound?

Partly

Are the conclusions drawn adequately supported by the results?

Partly

Are sufficient details of methods and analysis provided to allow replication by others?

Partly

Reviewer Expertise:

Bioenergy, biomass, biofuels, carbon dioxide biofixation, wastwe and wastewater management, air protection

I confirm that I have read this submission and believe that I have an appropriate level of expertise to confirm that it is of an acceptable scientific standard.

# Reviewer report: Reviewer response for version 2

**DOI:** 10.5256/f1000research.177927.r372543

**Published:** 2025-04-09

**Authors:** Rosangela Rodrigues Dias

**Affiliations:** 1 Federal University of Santa Maria, Santa Maria, Brazil

The manuscript addresses the environmental sustainability of microalgae-based products. Information regarding the analysis procedure and assumptions made is provided throughout the text. The work is technically sound, and the information provided allows for its replication and reproducibility. Some of the conclusions were already expected, for example, the benefit of integrating renewable energy into bioprocesses. However, the work elaborates on interesting scenarios, and topic 3.2 provides particularly valuable insights, especially considering that it is possible and economically viable to implement photovoltaic systems to meet the electricity demand of microalgae facilities.

 In sum, the manuscript raised doubts and questions that were clarified throughout the text. The article offers valuable contributions to microalgae biotechnology and represents a relevant addition to the scientific literature.

 After minor revisions, as detailed below, I recommend its acceptance.

 1. Mention the purity range of the CO₂ captured using the amine-based technology.

 2. The authors mention in the results that they considered emissions from amine-based technology in the final emissions balance. This is relevant information that should have been presented in the methodology.

 3. Please clarify whether the CO₂ injected and not captured and fixed was added to the emissions balance. That was not clear.

 4. Mention the biomass productivity that you considered in sections 2.2.1 and 2.2.2.

 5. In Figure 2, in the biodiesel scenario, I did not identify the "emissions" output. Please check.

Is the work clearly and accurately presented and does it cite the current literature?

Yes

If applicable, is the statistical analysis and its interpretation appropriate?

Not applicable

Are all the source data underlying the results available to ensure full reproducibility?

Yes

Is the study design appropriate and is the work technically sound?

Yes

Are the conclusions drawn adequately supported by the results?

Yes

Are sufficient details of methods and analysis provided to allow replication by others?

Yes

Reviewer Expertise:

Microalgae biotechnology; Environmental sustainability; Renewable energy; Life cycle assessment.

I confirm that I have read this submission and believe that I have an appropriate level of expertise to confirm that it is of an acceptable scientific standard.

# Response

**Published:** 2025-04-10

**Authors:** Shabbir Gheewala

**Affiliations:** King Mongkut's University of Technology Thonburi, Bangkok, Bangkok, Thailand

**Response to Reviewer**

 We would like to sincerely thank the reviewer for the thoughtful and constructive feedback. We appreciate the positive evaluation of our manuscript and the suggestions that helped enhance the clarity and completeness of our study. Below are our detailed responses to each point raised.

**Comments 1–3**

**Comment**:

1. Mention the purity range of the CO _2_ captured using the amine-based technology.
2. The authors mention in the results that they considered emissions from amine-based technology in the final emissions balance. This is relevant information that should have been presented in the methodology.
3. Please clarify whether the CO _2_ injected and not captured and fixed was added to the emissions balance. That was not clear.

**Response**:

 Thank you for these valuable comments. We have revised the **Methodology** section to address these points in detail. The following text has been added:

*“In this study, post-combustion CO _2_ capture is modeled using monoethanolamine (MEA) solvent, which is well-established and commonly applied in retrofitting existing fossil fuel power plants. The process involves absorbing CO _2_  from flue gases using MEA in an absorber column, followed by thermal regeneration of the solvent to release concentrated CO _2_. The recovered CO _2_ is then compressed and transported for downstream utilization. The captured CO _2_ itself is considered an emission stream and thus not assigned any environmental burden related to its formation. However, the energy and material inputs required for the capture process—including electricity and thermal energy consumption (approximately 378 kWh per tonne of CO _2_ captured), solvent use, and infrastructure—are fully accounted for and allocated to the system (Rafiq et al., 2024a; Win et al., 2023). This ensures that the environmental impacts of enabling CO _2_ capture are reflected in the lifecycle assessment. Amine-based CO _2_ capture typically produces CO _2_ with a purity >90% (Win et al., 2023). This high purity ensures effective uptake by the algal culture and avoids potential contamination. Importantly, while 100% of the captured CO _2_ is included in the system inventory, it is assumed that only 75% is fixed in the final products. The remaining 25% is modeled as released to the atmosphere, and thus contributes to the system’s overall emissions.*”

**Comment 4**:

*Mention the biomass productivity that you considered in sections 2.2.1 and 2.2.2.*

**Response**:

 Thank you for the suggestion. We have now included the relevant biomass productivity values in Section 2.2.1, which describes the microalgae cultivation conditions. The following text was added:

*“In this study, the microalgae are cultivated in borosilicate glass tubular photobioreactors, selected for their high surface area-to-volume ratio and efficient light utilization. The productivity of the system for three products is 0.69 g L⁻¹ d⁻¹ and a yield of 59 tonnes per hectare per year (Schade and Meier, 2020).*”

**Comment 5**:

*In Figure 2, in the biodiesel scenario, I did not identify the "emissions" output. Please check.*

**Response**:

 Thank you for bringing this to our attention. We have revised Figure 2 to clearly label the emissions output in the biodiesel scenario. This adjustment improves the clarity of the figure and ensures consistent representation of emissions across all scenarios.

# Reviewer report: Reviewer response for version 1

**DOI:** 10.5256/f1000research.174688.r352702

**Published:** 2025-01-10

**Authors:** Marcin Dębowski

**Affiliations:** 1 University of Warmia and Mazury, Olsztyn, Poland

In my opinion, the article raises an important issue of the use of micro-algae in the context of carbon sequestration (CCU) and its potential for the production of biofuels, fish feed and biofertilisers. The authors have used the life cycle analysis (LCA) method, which is suitable for assessing the environmental and economic impact of the proposed solutions. The article provides a fairly good basis for the analysis, but it lacks detailed references to existing literature and a more comprehensive comparison with the results of studies by other authors.

 In my opinion, the authors have set themselves the ambitious task of analysing three energy scenarios (TH20, 50% renewable energy sources, 100% renewable energy sources) and their impact on the production of three different products. The methodology is based on the ISO 14040 and 14044 standards, which is the main advantage of the work. This is always difficult with technologies that are not widely used in practise and the data are usually estimates from different, mainly experimental work. I believe that the choice of energy scenarios described and analysed was not sufficiently justified in the context of technical and economic possibilities. The authors should demonstrate the validity of the original assumptions more precisely and convincingly.

 In my opinion, the manuscript is not specify enough and partially ignores some important technological issues, such as the operating costs and efficiency of CCU integration on an industrial scale. It is also worth looking at the generalisability of these assumptions through the prism of climate policy in other regions of the world (Zieliński M, et al., 2023 [Ref 1])

 The conclusions and key messages could be strengthened and supported by reference to the results of other studies, particularly in the area of energy efficiency, technological efficiency and the cost of CCU technology. The data on micro-algae production could be more detailed, e.g. in relation to the efficiency of different photo-bioreactor systems, which would make the work more universal and valid for different climatic conditions.

 The paper does not address the economic aspects related to the implementation of CCU technology on an industrial scale, such as installation and operating costs, which can be completed based on the available literature. It would be useful to highlight the existing limitations in the widespread implementation of energy systems based on micro-algae biomass (Dębowski M, et al., 2022 [Ref 2])

Is the work clearly and accurately presented and does it cite the current literature?

Partly

If applicable, is the statistical analysis and its interpretation appropriate?

Not applicable

Are all the source data underlying the results available to ensure full reproducibility?

Partly

Is the study design appropriate and is the work technically sound?

Partly

Are the conclusions drawn adequately supported by the results?

Partly

Are sufficient details of methods and analysis provided to allow replication by others?

Partly

Reviewer Expertise:

biofuels production, algae cultivation, co2 emission reduction, biotechnology

I confirm that I have read this submission and believe that I have an appropriate level of expertise to confirm that it is of an acceptable scientific standard, however I have significant reservations, as outlined above.

## References

[1] : Microalgal Carbon Dioxide (CO2) Capture and Utilization from the European Union Perspective. *Energies* .2023;16(3) : 10.3390/en16031446 10.3390/en16031446

[2] : Large Scale Microalgae Biofuel Technology—Development Perspectives in Light of the Barriers and Limitations. *Energies* .2022;16(1) : 10.3390/en16010081 10.3390/en16010081

# Response

**Published:** 2025-02-03

**Authors:** Shabbir Gheewala

**Affiliations:** King Mongkut's University of Technology Thonburi, Bangkok, Bangkok, Thailand

The authors would like to thank the reviewer for kindly reading through the manuscript and providing expert comments which helped enhance the quality of our paper. The pointwise responses to the comments are provided below.

**Comment:**

*“The choice of energy scenarios described and analysed was not sufficiently justified in the context of technical and economic possibilities. The authors should demonstrate the validity of the original assumptions more precisely and convincingly.”*

**Response:**

 Thank you for your valuable comment. We have now clarified and expanded our discussion regarding the electricity scenarios for Thailand. The following amendment is proposed:

**"Electricity Scenarios for Thailand**

 Three electricity scenarios were evaluated based on anticipated changes in Thailand's energy mix. Thailand's current grid mix is predominantly fossil-based. According to the 2024 Power Development Plan, by 2037, the contribution of natural gas in the energy mix is expected to reduce to 41% from 57%, and coal is anticipated to drop to 7% from around 20%  ( Rafiq et al., 2024a). To explore the full potential of carbon capture and utilization pathways, a 100% renewable energy scenario is considered and modeled in SimaPro, reflecting these future energy shifts in Thailand."

**Comment:**

*“The article provides a fairly good basis for the analysis, but it lacks detailed references to existing literature and a more comprehensive comparison with the results of studies by other authors.”*

**Response:**

 Thank you for highlighting this issue. In response, we have expanded our literature review to include a more comprehensive comparison with previous studies.  The following amendment is proposed:

**"Comparison with Literature**

 While direct comparisons with previous studies are challenging due to differences in methodologies, system boundaries, and assumptions, existing literature provides insights into the potential of microalgae-based products for emission reduction. Microalgae-based biodiesel has consistently shown promising potential for reducing greenhouse gas emissions when compared to conventional fossil fuels. A conceptual design proposed by Martín and Grossmann (2017) suggests that integrating renewable energy sources into microalgae cultivation and processing could capture approximately 4 kg of CO _2_ for each kilogram of biodiesel produced, further highlighting the environmental advantages. In the same vein, recent study reported that biodiesel derived from microalgae could achieve up to 80% reductions in life cycle GHG emissions relative to petroleum-based diesel (Saranya and Ramachandra, 2020). Most recently, the work by (Xia et al., 2024) reinforces these findings, showing that freshwater- and wastewater-based cultivation can avoid 5.7 tonne CO _2_ eq and 6.8 tonne kg CO _2_ eq per tonne of biodiesel, respectively, highlighting the critical role water source selection and cultivation methods play in amplifying carbon capture and emissions reductions.

 Beyond fuel applications, microalgae offer significant environmental benefits in the food and feed sectors due to their nutrient-rich biomass, which can reduce reliance on conventional agricultural products. Smetana et al. (2017) highlighted that microalgae-derived protein powders, when optimized, can have lower environmental impacts than traditional animal-based proteins, making them a sustainable alternative for addressing global food demands. The potential of microalgae-based biofertilizers has also been explored for its environmental merits, although findings remain mixed. de Siqueira Castro et al. (2020) revealed that while microalgae-based biofertilizers offer potential for sustainable agriculture, they currently have higher environmental impacts than triple superphosphate, with significant contributions to climate change (3.17 kg CO _2_ eq) and terrestrial ecotoxicity (87.33% higher). The energy-intensive processes of cultivation, concentration, and drying were key contributors, requiring 6.51 kWh per gram of phosphorus. However, adopting renewable energy sources and natural drying methods could reduce these impacts, with a 47% reduction in climate change potential (de Siqueira Castro et al., 2020). Similarly, Chiew et al. (2015) reported that digestate-based fertilizers from anaerobic digestion of food waste typically have a greater environmental impact than chemical fertilizers when assessed through life cycle analysis metrics such as global warming, acidification, and eutrophication. However, digestate fertilizers demonstrate an advantage in reducing the reliance on non-renewable phosphate rock. Challenges for digestate include significant emissions, such as methane during digestion and ammonia during storage and application. While system improvements such as better methane capture could reduce the global warming of digestate, its higher acidification and eutrophication impacts remain a notable drawback​ (Chiew et al., 2015)."

**Comment:** *The paper does not address the economic aspects related to the implementation of CCU technology on an industrial scale, such as installation and operating costs, which can be completed based on the available literature.*

*The manuscript is not specific enough and partially ignores some important technological issues, such as the operating costs and efficiency of CCU integration on an industrial scale.*

**Response**: Thank you for emphasizing the importance of the economic perspective. We have now added a detailed discussion on the economic aspects, addressing both installation and operating costs for CCU technology. The revised manuscript now presents additional data (see Table S2 and Figure S1) that outline the levelized costs of different microalgae-based products. For example, while biodiesel production shows high capital and operating expenditures, the economic prospects for biofertilizer and fish feed are comparatively more favorable. We have also incorporated insights from Zieliński et al. (2023) and Dębowski et al. (2022) to better illustrate the limitations and opportunities for scaling microalgae-based energy systems.  The following amendment is proposed:

**"Constraints of Microalgae-Based Carbon Capture and Utilization**

 The deployment of microalgae for carbon capture and utilization is often limited by the availability and quality of renewable energy inputs. The share of renewables was also relatively low, mainly due to the relatively windless and low solar irradiation conditions in certain regions (Zieliński et al., 2023). For solar energy, the global average radiation intensity suitable for production is around 4.5 kWh/m ^2^/day, with approximately 208 regions falling into the optimal range of 4 to 5.5 kWh/m ^2^/day, including countries like the United States, China, Italy, and India (Maghzian et al., 2025). Thailand's solar energy potential is promising in this context, with an average daily radiation of 5.06 kWh/m ^2^/day, exceeding the global average, and reaching up to 5.17 kWh/m ^2^/day in Pattani Province and 5.08 kWh/m ^2^/day in Uthai Thani Province, making it highly suitable for solar-based production systems (Peerapong, 2021; RatedPower, 2022).

 Meeting 30% of global transportation energy needs by 2050 using renewable biofuels, such as microalgae, would require approximately 1 million km ^2^ of land, which constitutes about 1.1% of the Earth’s land area (Du et al., 2023; Maghzian et al., 2025). In Thailand alone, utilizing 47 million tonnes of CO _2_ through microalgae production would require approximately 1,500 km ^2^ of land, a value estimated based on Chisti (2007), which considers land requirements for flat-plate photobioreactors (Chisti, 2007).

 Despite such potential, microalgae-based CO _2_ utilization faces multiple constraints. Cultivation depends on maintaining specific pH, temperature, light intensity, and nutrient availability to sustain optimal photosynthetic rates (Ighalo et al., 2022). Economic viability is also limited by high energy requirements for mixing, harvesting, and downstream processing (Subhash et al., 2022). Microalgal species vary widely in their carbon dioxide tolerance, with some thriving in high CO _2_ levels while others struggle in acidic conditions, making species selection crucial for effective carbon capture (Solovchenko and Khozin-Goldberg, 2013; Zhu et al., 2017). Scaling faces additional challenges from insufficient pilot-scale data for reliable LCA and limited funding for innovation (Dębowski et al., 2023). While government incentives and regulations could reduce commercialization risks  (Chen et al., 2020; De Mendonca et al., 2021; Johansson and Kriström, 2019), intersectoral conflicts and potential negative impacts of renewable subsidies on economic welfare present ongoing challenges (Bashir et al., 2022; Dębowski et al., 2023; Johansson and Kriström, 2019).

 Table S2 clearly illustrates that microalgae-based production pathways require substantial capital investment, mainly driven by the photobioreactor infrastructure. Further analysis in Figure S1 demonstrates the levelized costs of different microalgae-based products, revealing that biodiesel production (5.5 USD/liter), with its high capital and operating expenditures, will need significant incentives to become commercially viable. On the other hand, both biofertilizer and fish feed show more competitive market-level costs, indicating their potential to be viable without relying heavily on subsidies. However, to fully assess their economic benefits, further field-scale data are required, and pilot-scale testing will be essential to refine these estimates and understand their real-world applicability."

**Comment:**

*“The conclusions and key messages could be strengthened and supported by reference to the results of other studies, particularly in the area of energy efficiency, technological efficiency and the cost of CCU technology.”*

**Response:**

 Thank you for your suggestion. To strengthen our conclusions and key messages, we have enhanced the discussion as follows. We have not added references in this section as it is rather uncommon to do so; we hope the reviewer may kindly accept our consideration.  The following amendment is proposed:

 "Furthermore, our analysis addresses the economic and technological challenges associated with scaling up carbon capture and utilization (CCU) technology. Although biodiesel production requires considerable capital and operating expenditures, the competitive cost profiles of fish feed and biofertilizer production offer attractive economic alternatives. Targeted policy support and continued technological innovation will be essential to enhance both the cost-effectiveness and scalability of microalgae-based CCU systems."

 Bashir, M. F., Ma, B., Bashir, M. A., Radulescu, M., Shahzad, U., 2022. Investigating the role of environmental taxes and regulations for renewable energy consumption: evidence from developed economies. Economic Research-Ekonomska Istraživanja. 35, 1262-1284. https://doi.org/10.1080/1331677X.2021.1962383.

 Chen, H., Wang, X., Wang, Q., 2020. Microalgal biofuels in China: The past, progress and prospects. Gcb Bioenergy. 12, 1044-1065. https://doi.org/10.1111/gcbb.12741.

 Chiew, Y. L., Spångberg, J., Baky, A., Hansson, P.-A., Jönsson, H., 2015. Environmental impact of recycling digested food waste as a fertilizer in agriculture—A case study. Resources, Conservation Recycling. 95, 1-14. https://doi.org/10.1016/j.resconrec.2014.11.015.

 Chisti, Y., 2007. Biodiesel from microalgae. Biotechnology advances. 25, 294-306. https://doi.org/10.1016/j.biotechadv.2007.02.001.

 De Mendonca, H. V., Assemany, P., Abreu, M., Couto, E., Maciel, A. M., Duarte, R. L., dos Santos, M. G. B., Reis, A., 2021. Microalgae in a global world: new solutions for old problems? Renewable Energy. 165, 842-862. https://doi.org/10.1016/j.renene.2020.11.014.

 de Siqueira Castro, J., Calijuri, M. L., Ferreira, J., Assemany, P. P., Ribeiro, V. J., 2020. Microalgae based biofertilizer: A life cycle approach. Science of the total environment. 724, 138138. https://doi.org/10.1016/j.scitotenv.2020.138138.

 Dębowski, M., Świca, I., Kazimierowicz, J., Zieliński, M., 2023. Large scale microalgae biofuel technology—development perspectives in light of the barriers and limitations. Energies. 16, 81. https://doi.org/10.3390/en16010081.

 Du, Y., Cheng, Q., Qian, M., Liu, Y., Wang, F., Ma, J., Zhang, X., Lin, H., 2023. Biodegradation of sulfametoxydiazine by Alcaligenes aquatillis FA: Performance, degradation pathways, and mechanisms. Journal of Hazardous Materials. 452, 131186. https://doi.org/10.1016/j.jhazmat.2023.131186.

 Ighalo, J. O., Dulta, K., Kurniawan, S. B., Omoarukhe, F. O., Ewuzie, U., Eshiemogie, S. O., Ojo, A. U., Abdullah, S. R. S., 2022. Progress in microalgae application for CO2 sequestration. Cleaner Chemical Engineering. 3, 100044. https://doi.org/10.1016/j.clce.2022.100044.

 Johansson, P.-O., Kriström, B., 2019. Welfare evaluation of subsidies to renewable energy in general equilibrium: Theory and application. Energy Economics. 83, 144-155. https://doi.org/10.1016/j.eneco.2019.06.024.

 Maghzian, A., Aslani, A., Zahedi, R., 2025. Analysis of suitable regions for microalgae cultivation and harvesting potential for carbon capture: A global feasibility study. Aquaculture. 595, 741496. https://doi.org/10.1016/j.aquaculture.2024.741496.

 Martín, M., Grossmann, I. E., 2017. Optimal integration of a self sustained algae based facility with solar and/or wind energy. Journal of Cleaner Production. 145, 336-347. https://doi.org/10.1016/j.jclepro.2017.01.051.

 Peerapong, P., 2021. Performance evaluation and the returns of investment of different solar PV panel types in the utility-scale: Cases of the installation in multi-region in Thailand. RMUTSB Academic Journal. 9, 174-185.

 RatedPower, 2022. Solar energy market in Thailand. Available online: https://ratedpower.com/blog/solar-energy-thailand/?utm_source

 Saranya, G., Ramachandra, T., 2020. Life cycle assessment of biodiesel from estuarine microalgae. Energy Conversion Management: X. 8, 100065. https://doi.org/10.1016/j.ecmx.2020.100065.

 Smetana, S., Sandmann, M., Rohn, S., Pleissner, D., Heinz, V., 2017. Autotrophic and heterotrophic microalgae and cyanobacteria cultivation for food and feed: life cycle assessment. Bioresource technology. 245, 162-170. https://doi.org/10.1016/j.biortech.2017.08.113.

 Solovchenko, Khozin-Goldberg, 2013. High-CO2 tolerance in microalgae: possible mechanisms and implications for biotechnology and bioremediation. Biotechnol Lett. doi: 10.1007/s10529-013-1274-7.

 Subhash, G. V., Rajvanshi, M., Kumar, G. R. K., Sagaram, U. S., Prasad, V., Govindachary, S., Dasgupta, S., 2022. Challenges in microalgal biofuel production: A perspective on techno economic feasibility under biorefinery stratagem. Bioresource Technology. 343, 126155. https://doi.org/10.1016/j.biortech.2021.126155.

 Xia, R., Liu, Y., Wang, Y., Zhao, Y., Ju, M., 2024. Life Cycle Environmental Impact Assessment of Microalgae Biodiesel Derived from Different Processes. Available at SSRN 4744668. http://dx.doi.org/10.2139/ssrn.4744668.

 Zhu, B., Chen, G., Cao, X., Wei, D., 2017. Molecular characterization of CO2 sequestration and assimilation in microalgae and its biotechnological applications. Bioresource Technology. 244, 1207-1215. https://doi.org/10.1016/j.biortech.2017.05.199.

 Zieliński, M., Dębowski, M., Kazimierowicz, J., Świca, I., 2023. Microalgal carbon dioxide (CO2) capture and utilization from the European Union perspective. Energies. 16, 1446. https://doi.org/10.3390/en16031446.
